# Diphtheria and Scarlet Fever

**Published:** 1897-04

**Authors:** G. E. Daly

**Affiliations:** NEW YORK.


ARTICLE VIII.

### DIPHTHERIA AND SCARLET FEVER.

BY G. E. DALY, M. D., NEW YORK.

The writer desires to report a series of twenty consecutive cases of diphtheria which have come under his care since May 1st, 1896. The twenty cases referred to occurred in seventeen different households. All of the affected were children, the oldest being twelve years of age, and the youngest slightly over a year. The latter child was suckled occasionally by its mother, but at other times, and more frequently, was fed from a bottle. The average age of the affected children was two years and six months.

All presented the clinical evidences (membrane, etc.) of diphtheria, and the diagnosis in each case was confirmed by bacteriological examination of the health department.

The children were, with one exception, seen on the first or second day of the appearance of the membrane. The case excepted was first seen on the seventh day of the disease.

In nineteen of the cases the plan of treatment pursued was as follows: namely, to administer to the sick children one-tenth-grain doses of calomel every hour until a fæcal evacuation had occurred, in the mean time giving every two hours liberal doses of ordinary iron, chlorate of potassium, and glycerin mixture, and constantly applying, when the child would permit, hot flaxseed poultices to the exterior of the throat.

Further, the health department had been notified of the existence of the disease, and each sick child, as well as the non-affected children of the same household, was injected by the health department, at the solicitation of the writer, with antitoxin.

The nineteen diphtheretic patients so treated promptly recovered, though some had been affected by the severe type of the disease, having extensive exudations, etc.

There were no resulting or accompanying complications in any of the cases, excepting for a slight pharyngeal paralysis in a boy of eight years.

The twentieth case, being the second of this series, was first seen after the disease had been in existence one week. In this child's throat a very general naso-pharyngeal and palatal exudation existed, and the child was in a nearly unconscious condition as the result of exhaustion and sepsis. The case being regarded as hopeless, antitoxin was not injected, though the other measures of treatment were carried out, but without avail, as the child died thirty-six hours after the beginning of treatment. It is probable that even had antitoxin been injected the child's life would not have been saved.

The writer has observed absolutely none of the ill effects that are said frequently to follow the use of antitoxin, although he has had under observation not only the nineteen sick children in whom the remedy was used, but also twenty-four other children, members of the same families or of disease-stricken households, to whom immunizing injections of antitoxin were administered.

In support of the immunizing power of antitoxin, the writer would say that in only three households did second cases of diphtheria develop, and in two of these three cases the children who became secondarily affected were feverish and apparently already infected by the diphtheritic process before antitoxin had been used upon them. From these two examples it may be inferred that injections of antitoxin have not the power of aborting diphtheria once the infection has occurred, although slight fever may be the only evidence of the approaching disease.

The third case gives this history: On July 5, 1896, Ida C——, aged ten years, was found suffering from a marked naso-pharyngeal diphtheria. Antitoxin, etc., was used for its treatment, and the other children of the household were ordered immunized. The girl recovered without complications. On July 27th (same month) her brother, aged twelve years, was reported sick and found to be suffering from diphtheria. Upon inquiry it was learned that when the physician of the health department had on July 6th injected the other members of the family with immunizing doses of antitoxin, this boy had run off, and had therefore not been so injected, but, as above recorded, later developed diphtheria, the only secondary case that occurred, or has since occurred in that household of seven children.

From such a history a possible power of immunization from diphtheria might reasonably be attributed to antitoxin injections.

The day following the writing of the preceding paragraph, one of the children referred to as having recovered from diphtheria became sick, and after ailing for twenty-four hours developed a fever of 100° F., pulse 120, with some suffusion of the eyes, and a rash, which appeared first on the face and later on the upper and lower extremities, but not at all upon the body. It was rose colored, like that of measles, and had a distal bordering of small and discrete papules, which proximally were preceded by patches as large as a half-dollar, and these by patches which had become confluent and erythematous areas raised wheal-like above the plane of the surrounding integument. There was no itching, as in urticaria; the lateral neck muscles were fixed and rigid, and the right knee-joint was much inflamed and painful as in rheumatism. The child had never had measels.

The question presented itself as to whether the child was developing rubeola or suffering from an urticaria, such as has been been reported as resulting from antitoxin injections, though in this child antitoxin had been injected but once, and that two weeks before the development of the rash in question, which latter had become manifest after a seemingly prodromal period of not more than twenty-four hours' duration. A positive diagnosis was not made. Salicylate of sodium was prescribed, and on the following day all of the rash and rheumatic symptoms had disappeared and have not since recurred. Rubela could not have been so aborted; hence the rash, so extensive but of so temporary a character, was probably uticarial.

The question still remains as to whether it and the joint symptoms, simulating an infection of some kind, were caused by the injection of antitoxin fourteen days before, or whether they arose from some other cause.

And now referring to scarlatina. There frequently occurs in scarlatina a type of sore throat, the severity of which seems not at all to depend upon the grade of the febrile disorder, in that the throat manifestations may be quite extensive and accompanied by but a slight fever, a fleeting rash, etc.

The throat disease in question is of an exudative (?) character, and may be seen upon the ulva, soft palate, tonsils, or posterior pharynx. It has all of the usual appearances of a diphtheritic membrane, and with scarlet fever is undoubtedly the condition so commonly termed "scarlet fever and diphtheria."

The writer, having remarked for some years that when this so-called association of scarlet fever and diphtheria occurred the affected children almost invariably recovered was at a loss to understand why, when the mortality of diphtheria alone averaged from thirty to fifty per cent. of those affected, that of the same disease complicating scarlet fever should have a so much lower mortality rate.

Accordingly since the first of the year, the writer, with the assistance of the health department, has had cultures made from the throats of all patients having scarlet fever in whom the throat exudations were such as would have led one, knowing nothing of the existing scarlet fever, to have diagnosed the throat condition as that of diphtheria. In all such cases, the invariable bacteriological report has been that no bacilli of diphtheria were found, but that there were found bacilli of a variety resembling those of diphtheria. If but one or two such reports had been received, considerable doubt might have existed as to whether the bacilli mentioned were not actually those of diphtheria; but as several such reports has been received, and, as already mentioned, the mortality for the conjoined (?) diseases is much lower than that of the more serious when not so associated, the writer has become somewhat doubtful as to the actual occcurrence of both diseases at one time and in the same patient.

In other words, here are two diseases: one serious, the other more so, with death resulting frequently from either, and yet the attention of the physician is frequently invited to the inspection of children who are said to have a simultaneous seizure of both.—*Medical Record.*

